# [^18^F]mFBG PET/CT surpasses [^18^F]FDG PET/CT for evaluation of pediatric neuroblastoma

**DOI:** 10.3389/fonc.2025.1627403

**Published:** 2025-09-02

**Authors:** Wenqian Zhang, Chao Yang, Zhenzhen Zhao, Shunhao Zhou, Yanyao Han, Chenxi Zheng, Yalan Xiong, Changchun Li, Yao Zhang, Zhenni Wang, Liang Cai, Shan Wang

**Affiliations:** ^1^ Department of Nuclear Medicine, The Second Affiliated Hospital of Chongqing Medical University, Chongqing, China; ^2^ Department of Surgical Oncology, National Clinical Research Center for Child Health and Disorders, Ministry of Education Key Laboratory of Child Development and Disorders, China International Science and Technology Cooperation Base of Child Development and Critical Disorders, Children’s Hospital of Chongqing Medical University, Chongqing, China

**Keywords:** [^18^F]mFBG, [^18^F]FDG, norepinephrine transporter, neuroblastoma, PET/CT

## Abstract

**Purpose:**

[^18^F]FDG PET/CT serves as an alternative imaging modality for neuroblastoma in cases where [^123^I]MIBG yields negative results or is unavailable. [^18^F]mFBG, a novel PET tracer for neuroblastoma imaging, requires further clinical validation. This preliminary study aims to evaluate the efficacy of [^18^F]mFBG PET/CT compared to [^18^F]FDG PET/CT in detecting neuroblastoma.

**Methods:**

In this retrospective investigation, 56 pediatric patients were enrolled. Each patient underwent both [^18^F]mFBG PET/CT and [^18^F]FDG PET/CT within one week. Two children underwent a second paired [^18^F]FDG-[^18^F]mFBG PET/CT scan. In total, 58 paired scans (mean age 47.6 ± 38.0 months, range 6–108 months) were performed. Two experienced readers measured normal organ uptake (SUV_mean_), lesion uptake (SUV_max_), and tumor-to-background ratio (TBR). A lesion-by-lesion analysis was conducted to compare detection rates between [^18^F]mFBG and [^18^F]FDG.

**Results:**

Twenty paired scans exhibited negative findings on both [^18^F]mFBG and [^18^F]FDG studies. Among the remaining 38 scans, 8 (21.05%) were [^18^F]mFBG-positive/[^18^F]FDG-negative, 1 (2.63%) was [^18^F]FDG-positive/[^18^F]mFBG-negative, and 29 (76.32%) were positive on both tracers. In these 38 scans, [^18^F]mFBG PET/CT identified 431 lesions, whereas [^18^F]FDG PET/CT detected only 162 lesions (p<0.001). Six of eight [^18^F]mFBG-positive/[^18^F]FDG-negative cases were histopathologically confirmed as neuroblastoma. The mean TBR of [^18^F]mFBG PET/CT(6.68 ± 5.76) was significantly higher (p<0.001) than that of [^18^F]FDG PET/CT (4.49 ± 2.88).

**Conclusion:**

[^18^F]mFBG PET/CT detected more neuroblastoma lesions than [^18^F]FDG PET/CT, suggesting it may be a more viable alternative when standard [^123^I]MIBG scanning is not feasible.

## Introduction

Neuroblastoma is a malignant solid tumor arising from neural crest, accounting for approximately 8% of pediatric cancers and nearly 15% of cancer-related fatalities in children. Approximately 50% of patients present with soft and/or distant skeletal metastases at diagnosis, a key prognostic factor for poor outcome (1, 2). The 5-year event-free survival rate is typically less than 50% (3, 4), underscoring the critical importance of precise staging for optimal treatment planning and clinical monitoring.

The norepinephrine transporter (NET), a transmembrane protein responsible for norepinephrine reuptake, is highly expressed in neuroblastoma cells. Iodine-123-labeled *meta*-iodobenzylguanidine ([^123^I]MIBG), a synthetic norepinephrine analogue, is the most widely used radiopharmaceutical for NET imaging (5). Currently, [^123^I]MIBG planar whole-body scintigraphy remains the gold standard for neuroblastoma nuclear imaging (6–8). However, [^123^I]MIBG scintigraphy has several limitations, including a 2-day imaging protocol, the need for thyroid blockade, limited spatial resolution, and frequent requirement for procedural sedation during scanning (8, 9). Moreover, only four hospitals in China currently have access to [^123^I]MIBG.

Positron emission tomography (PET) offers significant advantages over conventional scintigraphy, including shorter scan time, superior sensitivity, higher spatial resolution, and more straightforward radiotracer uptake quantification (10). Several PET tracers have shown promise for neuroblastoma imaging, such as [^18^F]FDG, [^18^F]F-DOPA, [^124^I]MIBG, [^68^Ga]DOTATATE, with studies demonstrating their ability to detect more tumor lesions compared to paired [^123^I]MIBG scans (11–14). The NCCN guidelines recommend [^18^F]FDG PET/CT as second-line imaging for MIBG-negative neuroblastoma cases (7). In China, where [^123^I]MIBG remains largely unavailable, [^18^F]FDG PET/CT has become the de facto standard for evaluating neuroblastic tumors and metastatic sites. Studies have demonstrated the clinical utility of [^18^F]FDG PET/CT in neuroblastoma, reporting higher detection accuracy than [^123^I]MIBG scintigraphy in selected patient cohorts (15–17). Furthermore, multiple studies have investigated the prognostic relevance of [^18^F]FDG PET/CT in pediatric neuroblastoma cases (18–20). However, [^18^F]FDG has limited specificity for skeletal metastases due to high physiological bone marrow uptake, particularly following treatment (8).

*Meta*-[^18^F]fluorobenzylguanidine ([^18^F]mFBG), an MIBG analog, similarly targets NET-expressing cells (21). This tracer has demonstrated favorable safety profiles, biodistribution characteristics, kinetic properties, and lesion detection capability (22). Comparative studies have shown similar physiological and pathological distributions between [^18^F]mFBG PET/CT and [^123^I]MIBG scintigraphy, with the former offering superior spatial resolution, improved tumor delineation, and enhanced lesion detection (23–25). Nevertheless, in regions where [^123^I]MIBG is unavailable, it remains unclear whether [^18^F]mFBG PET/CT should be prioritized over [^18^F]FDG PET/CT as the preferred imaging modality.

This preliminary study aims to evaluate the diagnostic performance of [^18^F]mFBG PET/CT versus [^18^F]FDG PET/CT in neuroblastoma assessment, seeking to determine its potential as a superior imaging alternative.

## Materials and methods

### Study design and patients

This retrospective study was conducted following approval by the Institutional Review Board of XXX Hospital from December 2023 to August 2024. Pediatric neuroblastoma patients meeting the following inclusion criteria were enrolled: (a) histopathological confirmed neuroblastoma diagnosis; (b) age > 6 months; (c) legal guardian consent for study participation; (d) ability to complete both [^18^F]mFBG PET/CT and [^18^F]FDG PET/CT scans within a 7-day interval; and (e) no therapeutic interventions between scans. Exclusion criteria included pregnancy. Written informed consent was obtained from all legal guardians and from patients aged >8 years. The histopathological analysis was utilized as the primary diagnostic reference standard. For lesions inaccessible to biopsy, clinical follow-up data supplemented by conventional imaging (CT/MRI) as secondary reference standards were utilized.

### [^18^F]mFBG PET/CT and [^18^F]FDG PET/CT imaging

The synthesis method for [^18^F]mFBG has been previously documented (26). The imaging studies were performed utilizing a time-of-flight (TOF) and point spread function (PSF) PET/CT scanner (uMI780, Shanghai United Imaging Healthcare Co., LTD, China). Both radiotracers ([^18^F]mFBG and [^18^F]FDG) were administered via slow intravenous injection at a dose of 2 MBq/kg (minimum activity: 20 MBq), followed by saline flush,with no fasting requirement for [^18^F]mFBG imaging. PET acquisition commenced ≥60 minutes post-injection with a 2-minute acquisition per bed position (25), preceded by a low-dose CT scan (80–100 kV, 30–50 mAs) for attenuation correction (24, 25). Images reconstruction employed an ordered subsets expectation maximization algorithm (2 iterations, 10 subsets) with corrections for CT-based attenuation, decay, random events, and scatter, yielding final PET images with 1.5 mm slice thickness.

### Lesion detection and image interpretation

Two experienced nuclear medicine physicians (XXX and XXX) independently evaluated anonymized scans for pathological lesions, with discordant interpretations resolved through consensus discussion involving a third senior reader (XXX). Pathological neuroblastoma lesions were defined as focal areas of [^18^F]FDG or [^18^F]mFBG uptake in soft tissue or bone/bone marrow that exceeding both adjacent tissues and contralateral reference areas, while absence of such distinct focal activity was classified as negative (24, 27, 28). To minimize confounding factors in [^18^F]FDG interpretation, strict protocols were implemented (1): comprehensive prescan clinical assessments documenting surgical history, treatment timelines, and recent febrile/activity status; and (2) expert image analysis applying stringent diagnostic criteria that classified diffuse low-to-moderate bone marrow uptake as negative (especially during post-treatment recovery), with all findings clinically correlated. Quantitative analysis measured maximum/mean standardized uptake values (SUV_max/mean_) for both lesions and normal organ backgrounds, enabling comparison of [^18^F]mFBG and [^18^F]FDG detection performance. Tumor-to-background ratios (TBR) were calculated by dividing lesion SUV_max_ by site-specific background SUV_mean_ (liver for hepatic lesions, bone/bone marrow for bone/bone marrow lesions, gluteal muscle for other lesions) (24, 29).

### Statistical analysis

Owing to the low prevalence of neuroblastoma, the sample size recruited was determined by practical considerations. For continuous data, normality tests (Shapiro - Wilk test) were performed. Data meeting normal distribution criteria are now presented as mean ± standard deviation (SD), while non - normal data retain the median and range description. Differences in lesion detection per region and lesion uptake were analyzed using Mann - Whitney U test between [^18^F]mFBG PET/CT and [^18^F]FDG PET/CT studies. The McNemar test and Mann - Whitney U tests was employed to compare the diagnostic performance and TBR values of the two techniques, respectively. Statistical analyses were performed using SPSS software (version 26, SPSS Inc., Chicago, IL, USA). The statistical significance was defined as a p-value <0.05.

## Results

### Clinical characteristics of the participants

**Figure 1:**
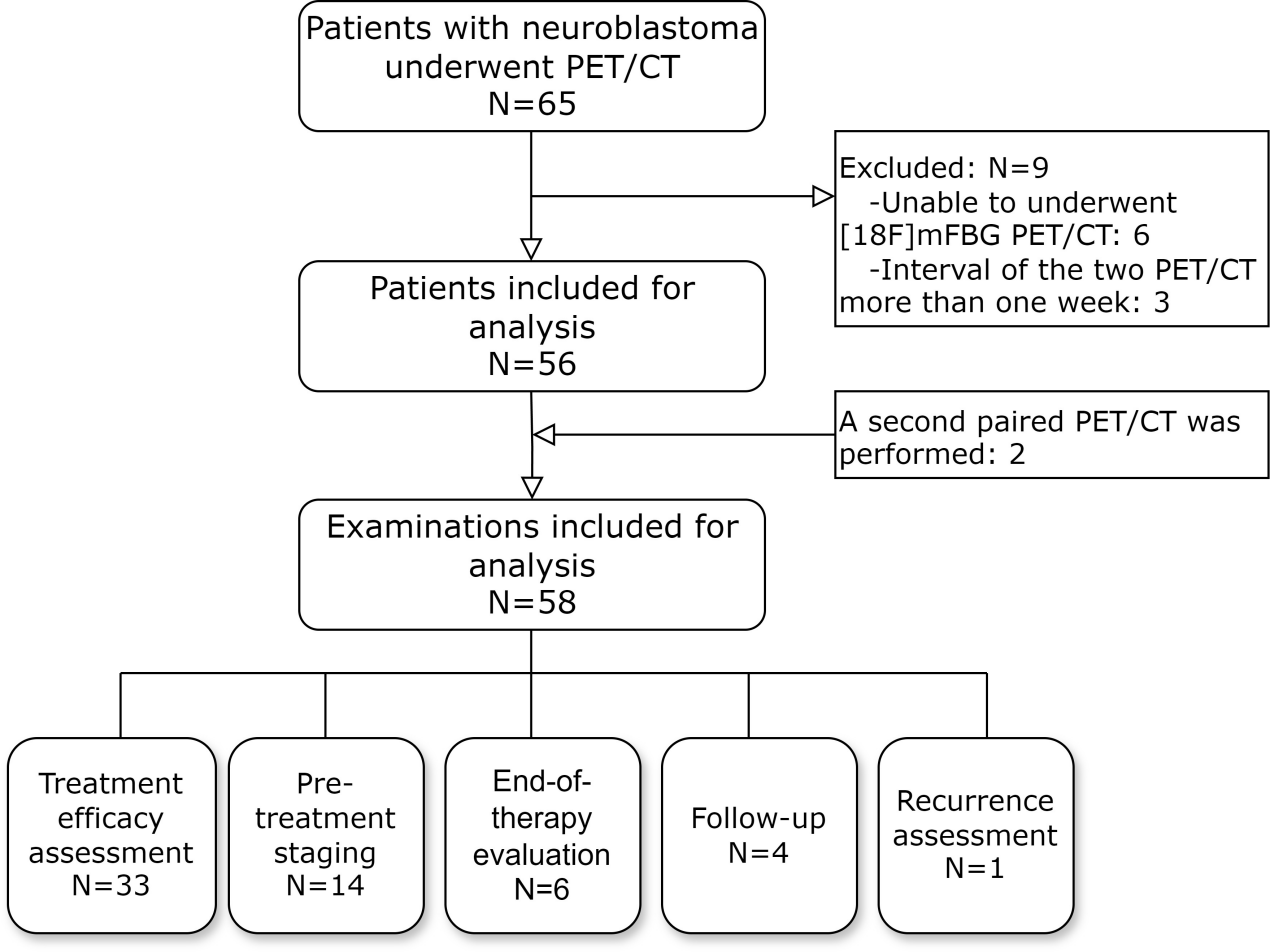
The flow diagram shows participant selection details.

Sixty-five pediatric patients with neuroblastoma who underwent PET/CT imaging were initially enrolled. After applying exclusion criteria, 56 patients (28 females and 28 males, mean age 47.6 ± 38.0 months, range 6–108 months) were included in the final analysis. Exclusion criteria comprised: 6 patients who failed to complete both [^18^F]mFBG and [^18^F]FDG PET/CT scans, and 3 patients whose paired scans exceeded the one-week interval requirement. Notably, two children underwent a second set of paired scans during treatment response assessment, resulting in a total of 58 analyzable [^18^F]FDG-[^18^F]mFBG PET/CT scan pairs. The clinical indications for scanning included: treatment response evaluation (33 scans, 56.89%), pretreatment staging (14 scans, 24.14%), end-of-therapy assessment (6 scans, 10.34%), follow-up monitoring (4 scans, 6.89%), and recurrence detection (1 scan, 1.72%) (**Figure 1**, **Table 1**).

**Table 1:** Patients and tumor characteristics.

Characteristics	[^18^F]mFBG PET/CT			[^18^F]FDG PET/CT		
	Positive (N=36)	Negative (N=20)	Total (N=56)	Positive (N=30)	Negative (N=26)	Total (N=56)
Age (months*)	50 ± 28.7 (9–106)	44.5 ± 27.3 (6–108)	47.6± 28.0 (6–108)	47.2 ± 28.9 (6–106)	46.1 ± 26.1 (12–108)	47.6± 28.0 (6–108)
Gender						
Male	20	8	28	18	10	28
Female	16	12	28	12	16	28
INRG Stage						
L1	10	1	11	9	2	11
L2	4	7	11	5	6	11
M	22	12	34	16	18	34
Risk						
Low	11	1	12	10	2	12
Intermediate	3	6	9	4	5	9
High	22	13	35	16	19	35
Indication for scan (58 paired scans)	Positive (N=37)	Negative (N=21)	Total (N=58)	Positive (N=30)	Negative (N=28)	Total (N=58)
Initial staging	14	0	14	14	0	14
Treatment efficacy assessment	20	13	33	14	19	33
EOT assessment	2	4	6	1	5	6
Follow-up	0	4	4	0	4	4
Recurrence	1	0	1	1	0	1

*Age is expressed as the mean± SD, with a range in parentheses. INRG, International Neuroblastoma Risk Group; EOT, end-of-therapy.

The primary tumor distribution analysis revealed 45 retroperitoneal cases, with remaining sites including mediastinum (n=4), pelvis (n=2), neck (n=2) and paravertebral/epidural area (n=2), and spermatic cord(n=1). Notably, the spermatic cord has not been previously reported as a primary site for neuroblastoma. According to the International Neuroblastoma Risk Group (INRG) staging system, 34 patients (60.71%) presented with stage M disease, while 35 patients (62.50%) were classified as high-risk. The median number of prior chemotherapy cycles at imaging was 6 (range 0-10).

No related adverse effects were observed following [^18^F]mFBG administration. Scan duration showed no significant difference between modalities ([^18^F]mFBG: 7.4 ± 2.8 min vs. [^18^F]FDG: 7.2 ± 3.0 min). Sedation was administered to 11 children during [^18^F]mFBG scans (mean age 1.8 ± 0.8 years) and 10 children during [^18^F]FDG scans (mean age 1.6 ± 1.0 years).

### Comparison of detection sensitivity between [^18^F]mFBG PET/CT and [^18^F]FDG PET/CT: patient-based analysis

**Figure 2:**
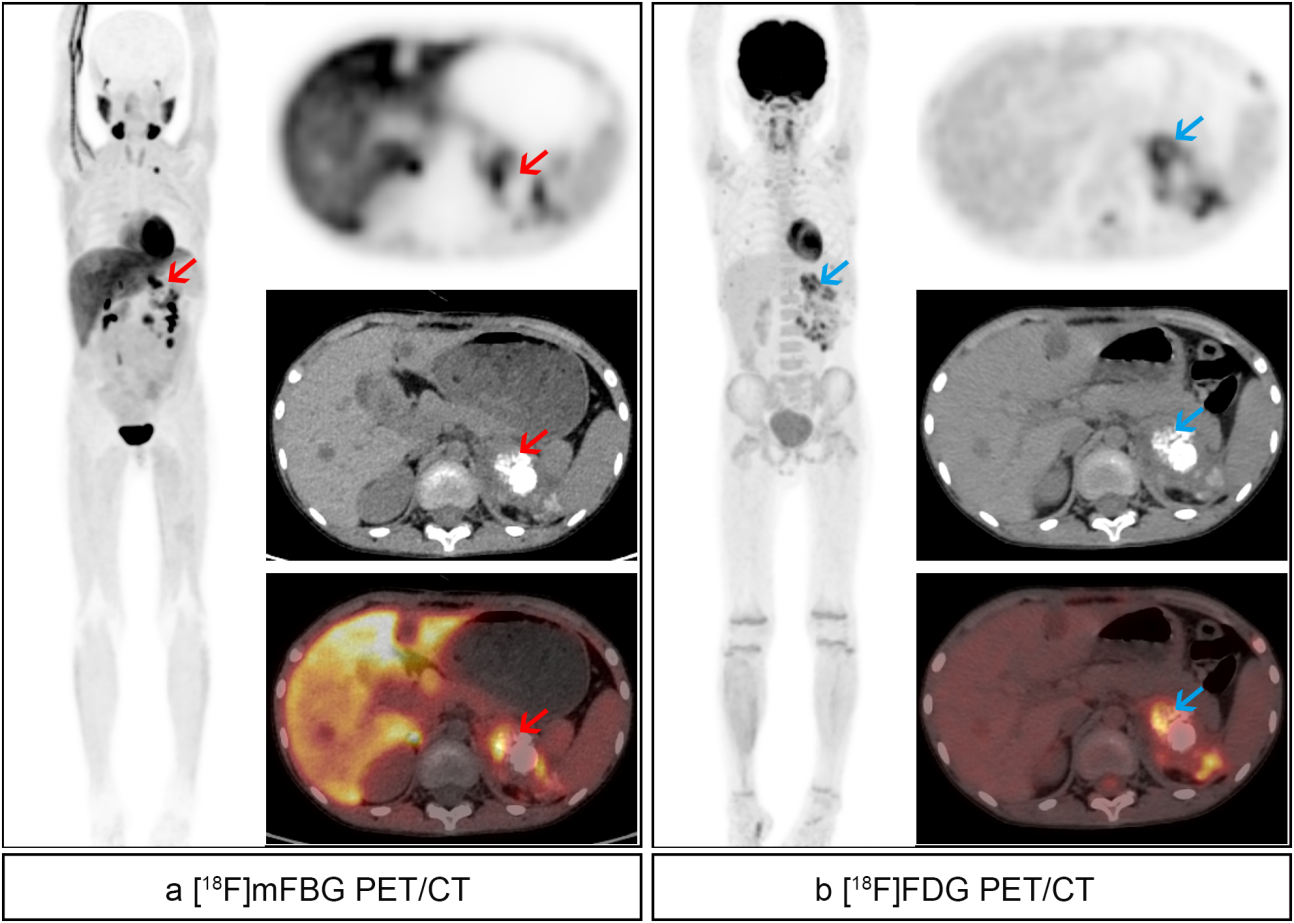
An 8-year-old boy with high-risk left retroperitoneal neuroblastoma received 1 cycle of chemotherapy. **(a)** [^18^F]mFBG images. Pathological uptake (red arrows) with SUVmax 8.9 and TBR 12.5 was noted in the left retroperitoneal mass, and detailed showed in axial images. **(b)** [^18^F]FDG images. Pathological uptake (blue arrows) with SUVmax 5.8 and TBR 11.9 was noted in the left retroperitoneal mass, and detailed show in axial images. Besides, residual activity of mFBG and FDG was found in the left thoracic entrance and retroperitoneal lymph nodes.

Twenty paired scans demonstrated concordant negative findings (13 treatment response evaluations, 4 EOT, and 3 follow-up scans), consistent with disease remission. Discordant results included 8 [^18^F]mFBG-positive/[^18^F]FDG-negative scans and 1 [^18^F]FDG-positive/[^18^F]mFBG-negative scan, while 29 scans showed positive on both modalities. The overall concordance rate between [^18^F]mFBG and [^18^F]FDG PET/CT was 84.48% (49/58) (representative examples shown in **Figure 2**).

Patient-based analysis revealed [^18^F]mFBG PET/CT detected significantly more soft tissue and skeletal lesions (63.79%, 37/58) compared to [^18^F]FDG PET/CT (51.72%, 30/58, p=0.039), with the difference being particularly pronounced in chemotherapy-treated patients. In the treatment response evaluation subgroup (n=33), [^18^F]mFBG demonstrated superior detection rates (60.60%, 20/33) versus [^18^F]FDG (42.42%, 14/33, p=0.031). However, no significant difference emerged between modalities (p=1.000) for initial staging (n=14), EOT evaluation (n=6), follow-up (n=4), or recurrence assessment (n=1) subgroups (combined detection rates: [^18^F]mFBG 68.00% [17/25] vs. [^18^F]FDG 64.00% [16/25]). Among 34 M-stage patients, [^18^F]mFBG maintained significantly higher positivity rates (64.70%, 22/34) than [^18^F]FDG (47.06%, 16/34, p=0.031).

**Figure 3:**
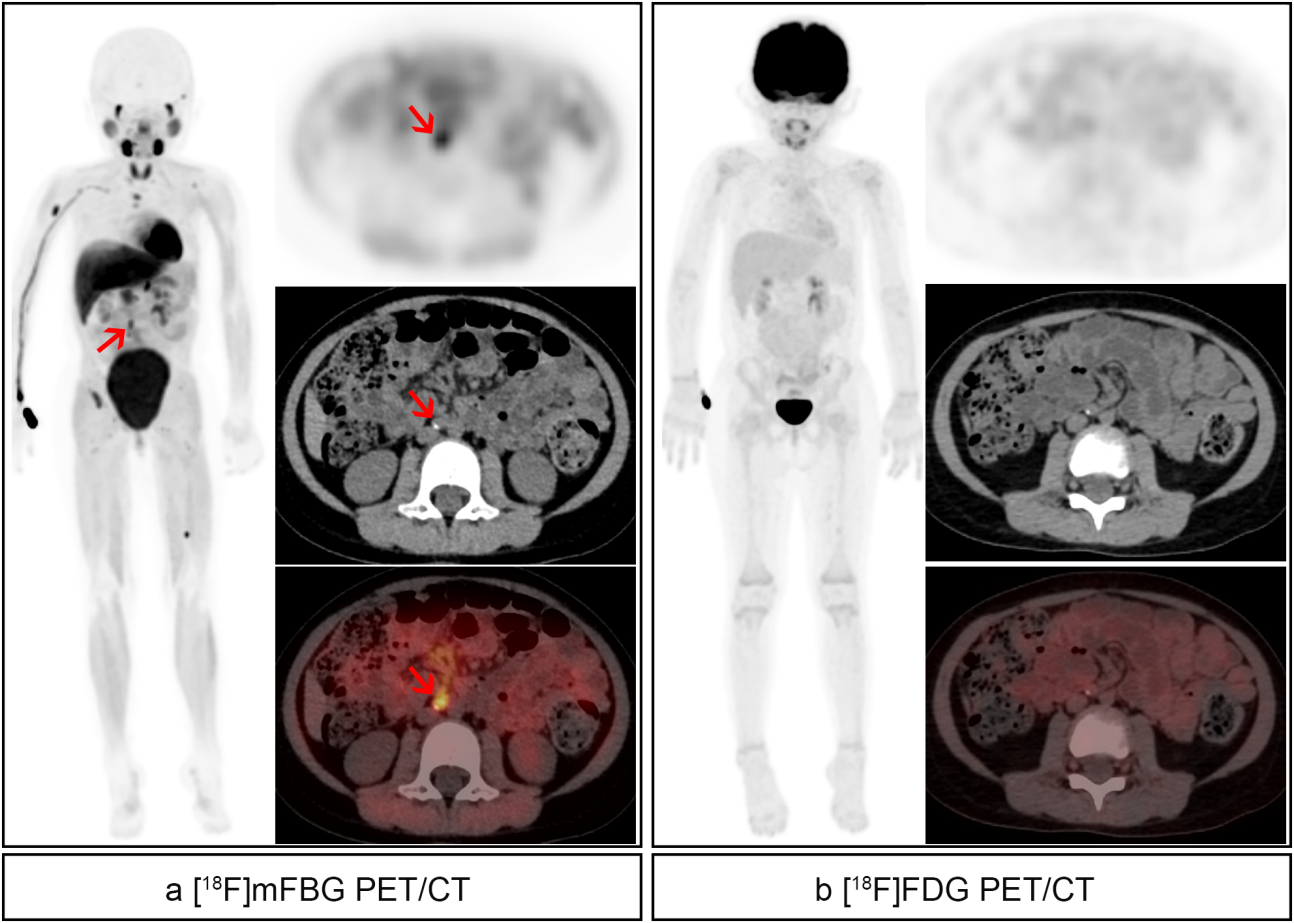
A 3-year-old girl suffered high-risk right retroperitoneal neuroblastoma with bone metastases and underwent resection of lesions followed by 8 cycles of chemotherapy. Post-therapy images were acquired. **(a)** [^18^F]mFBG images. Pathological uptake (red arrows) with SUVmax 5.1 and TBR 4.9 was noted in the abdominal paraaortic lymph nodes, and detailed showed in axial images. And residual activity of mFBG was found in bone/bone marrow in multiple parts of the body. **(b)** [^18^F]FDG images. No pathological uptake was found.

**Figure 4:**
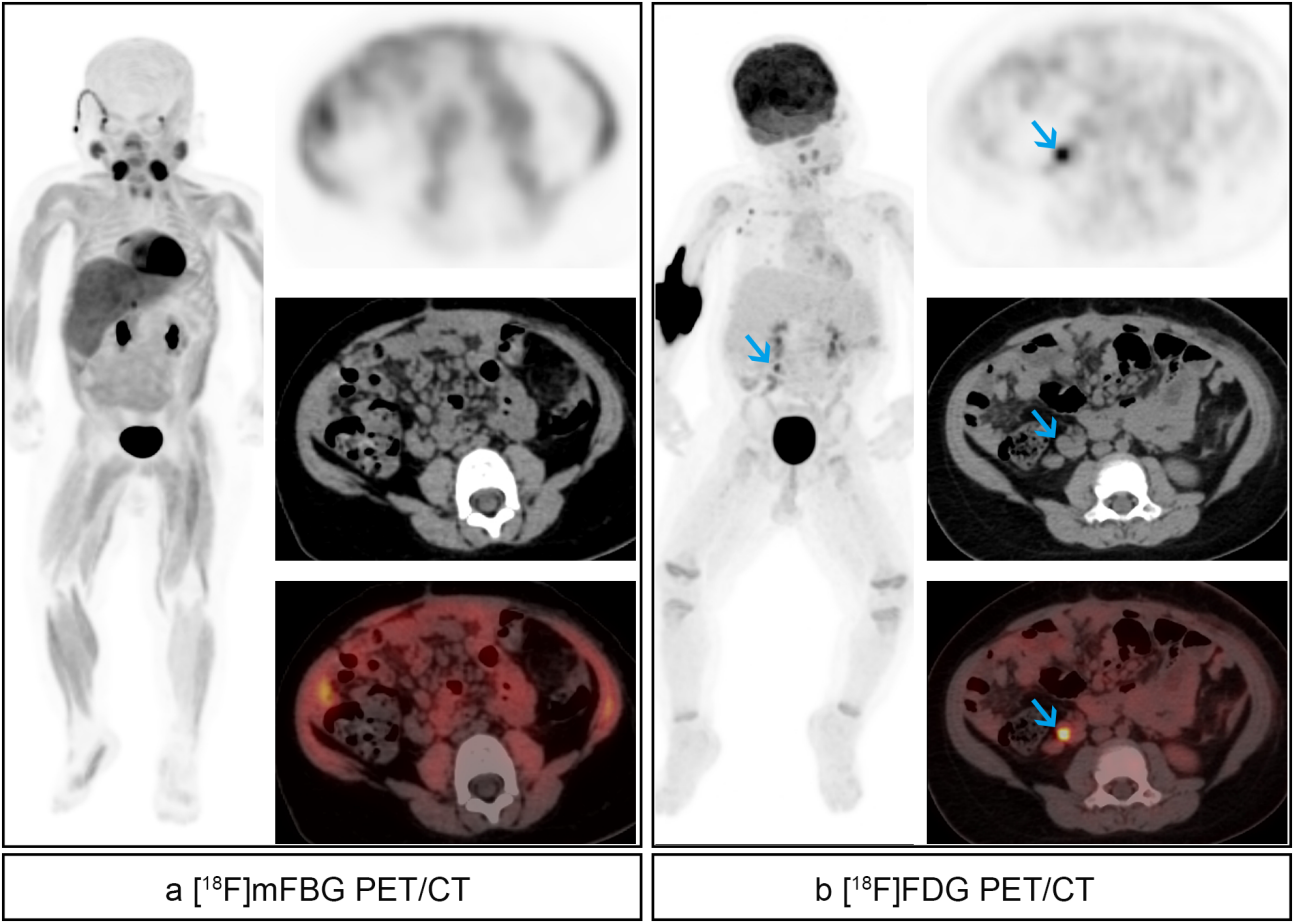
A 2-year-old boy suffered low-risk left retroperitoneal neuroblastoma and followed by 2 cycles of chemotherapy. Post-therapy images were acquired. **(a)** [^18^F]mFBG images. No residual activity was found. **(b)** [^18^F]FDG images. Abnormal activity (blue arrows) with SUVmax 5.0 and TBR 11.6 was found in the right retroperitoneal lymph node.

Histopathological confirmation was obtained for six of eight [^18^F]mFBG-positive/[^18^F]FDG-negative cases, all verifying active neuroblastoma (**Figure 3**). Conversely, the solitary [^18^F]FDG-positive/[^18^F]mFBG-negative cases showed no malignant cells on pathological examination (**Figure 4**).

### Lesion-based comparative analysis of [^18^F]mFBG and [^18^F]FDG PET/CT performance

**Figure 5:**
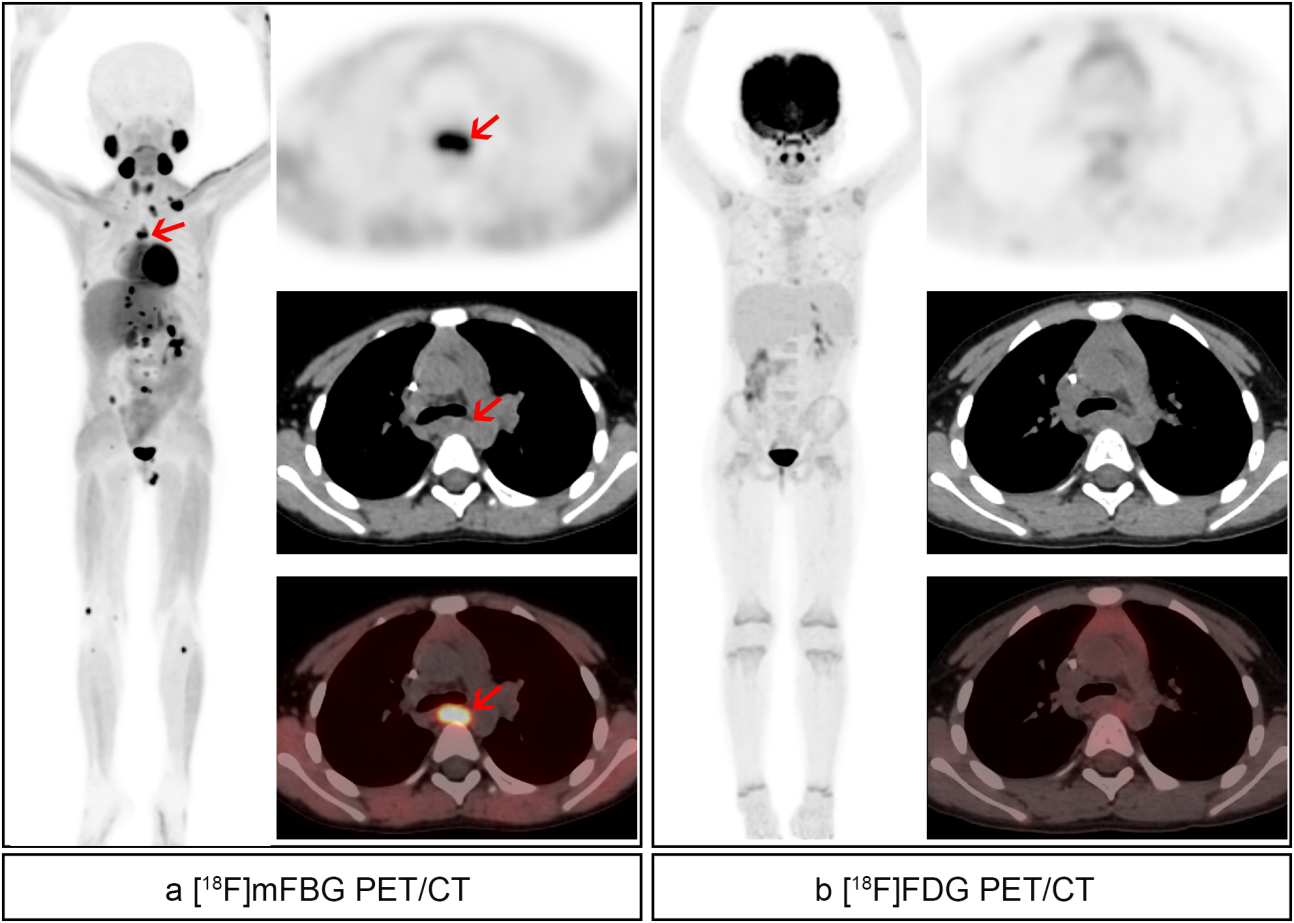
Therapy response evaluation of a 4-year-old boy suffered high-risk right retroperitoneal neuroblastoma underwent resection of lesions followed by 3 cycles of chemotherapy. **(a)** [^18^F]mFBG images. Pathological uptake was found in extensive lymph nodes and bone/bone marrow (red arrows). **(b)** [^18^F]FDG images. No residual activity was found.

The lesion-based evaluation demonstrated superior detection capability of [^18^F]mFBG PET/CT, which identified 431 total lesions (130 soft tissue, 301 skeletal) in 37 positive scans, compared to only 162 lesions (86 soft tissue, 76 skeletal) detected by [^18^F]FDG PET/CT in 30 positive scans (**Figure 5**). Among 29 scan pairs positive on both modalities, [^18^F]mFBG showed greater sensitivity for soft tissue lesions in 11 cases (37.93%), equivalent detection in 16 cases (55.17%), and reduced detection in 2 cases (6.89%), while for skeletal lesions it demonstrated superior detection in 23 cases (79.31%), comparable results in 4 cases (13.79%), and inferior performance in 2 cases (6.89%). Stratified analysis by INRG stage revealed comparable performance in early-stage (L1/L2) and low/intermediate-risk patients, while [^18^F]mFBG significantly outperformed [^18^F]FDG in metastatic (M stage) and high-risk groups (**Table 2**). Although both modalities detected more lesions at initial diagnosis and treatment response evaluation, only the efficacy assessment subgroup reached statistical significance.

**Table 2:** The number of lesions detected by two examination methods.

Characteristics	[^18^F]FDG PET/CT			[^18^F]mFBG PET/CT			P
	Soft tissues	BBM	Total	Soft tissues	BBM	Total	
INRG stage							
L1	14	0	14	14	0	14	P<1.00
L2	4	0	4	3	0	3	P<1.00
M	68	76	144	113	301	414	P<0.001
Risk group							
Low	15	0	15	15	0	15	P<1.00
Intermediate	3	0	3	2	0	2	P<1.00
High	68	76	144	113	301	414	P<0.001
Indication for scan							
Initial staging	28	9	37	30	34	64	0.18
Efficacy evaluation	51	67	118	94	265	359	0.001
EOT assessment	1	0	1	0	2	2	P<1.00
Follow-up	0	0	0	0	0	0	P<1.00
Recurrence	6	0	6	6	0	6	P<1.00

BBM, bone/bone marrow; INRG, International Neuroblastoma Risk Group; EOT, end of therapy.

Quantitative analysis showed significantly higher mean TBR for [^18^F]mFBG (6.68 ± 5.76) versus [^18^F]FDG (4.49 ± 2.88, p<0.001), with particularly notable differences in skeletal lesions ([^18^F]mFBG 6.72 ± 6.06 vs. [^18^F]FDG 3.26 ± 1.71, p<0.001) compared to soft tissue lesions ([^18^F]mFBG 6.56 ± 4.88 vs. [^18^F]FDG 5.71 ± 2.88, p=0.180).

### Clinical impact on therapeutic decision-making

The detection discrepancy between imaging modalities significantly influenced treatment strategies in three of eight [^18^F]mFBG-positive/[^18^F]FDG-negative cases, promoting therapeutic modifications involving surgical intervention followed by systemic therapy. The remaining five cases, while confirming skeletal metastases through [^18^F]mFBG imaging, maintained their original staging, risk classification, and treatment protocols.

A representative case involved a patient with primary right cervical neuroblastoma (initially staged as non-metastatic) whose post-operative [^18^F]mFBG PET/CT revealed previously undetected tibial metastases (upstaged to M), subsequently receiving chemotherapy, whereas concurrent [^18^F]FDG imaging only demonstrated expected inflammatory uptake at the surgical site. Conversely, the solitary [^18^F]FDG-positive/[^18^F]mFBG-negative case underwent parent-requested surgical excision that histopathologically confirmed the absence of malignant cells, validating the [^18^F]mFBG negative finding.

## Discussion

This prospective study evaluated the diagnostic performance of [^18^F]mFBG PET/CT in 56 neuroblastoma patients through 58 paired scans with [^18^F]FDG PET/CT, demonstrating excellent safety with no adverse reactions observed. Both modalities showed comparable acquisition times (approximately 7 minutes), consistent with prior reports (24, 25). Our findings reveal [^18^F]mFBG’s superior diagnostic sensitivity versus [^18^F]FDG in both patient-based (63.79% [37/58] vs. 51.72% [30/58], p=0.039) and lesion-based analyses (431 vs. 162 lesions detected, p<0.001). Quantitative assessment showed significantly higher mean TBR for [^18^F]mFBG (6.68 ± 5.76) compared to [^18^F]FDG (4.49 ± 2.88, p<0.001), with particularly notable differences in skeletal lesion detection. Pathological confirmation was obtained in seven surgically-treated cases (per parental request), with six [^18^F]mFBG-positive/[^18^F]FDG-negative lesions histologically verified as neuroblastoma, while the single [^18^F]FDG-positive/[^18^F]mFBG-negative case showed no malignant evidence. These results substantiated [^18^F]mFBG PET/CT’s superior specificity for post-treatment evaluation, suggesting its potential as a more reliable imaging biomarker than [^18^F]FDG PET/CT for therapeutic monitoring in neuroblastoma patients.

**Figure 6:**
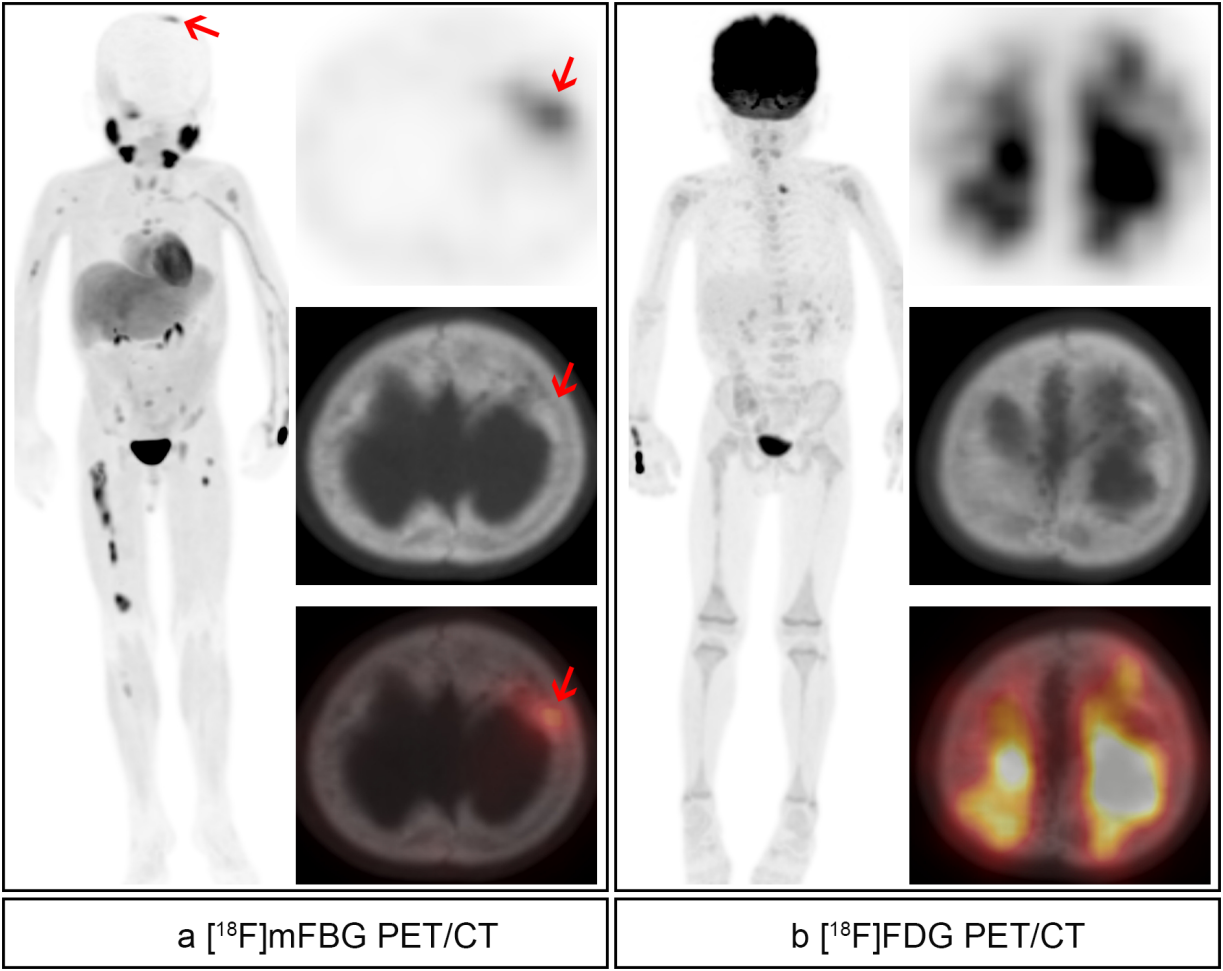
Therapy response evaluation of a 4-year-old boy with high-risk right retroperitoneal neuroblastoma underwent resection of lesions followed by 8 cycles of chemotherapy. **(a)** [^18^F]mFBG images. Pathological uptake was found in the skull (red arrows). **(b)** [^18^F]FDG images. No residual activity was found in the same site.

Our results demonstrate that [^18^F]mFBG PET/CT outperformed [^18^F]FDG PET/CT in detecting skeletal lesions, identifying 269 additional metastases, including skull lesions that were obscured by physiological [^18^F]FDG uptake (**Figure 6**). The statistically superior performance in treatment response assessment (compared to initial staging) likely reflects the predominance of early-stage disease (9 L1, 3 L2, and only 2 M stage cases) in our staging cohort, where lower lesion burden limited comparative sensitivity. Several methodological constraints must be acknowledged, including limited histopathological validation due to ethical and practical biopsy restrictions, lack of direct comparison with [^123^I]MIBG imaging, and reliance on CT/MRI and clinical follow-up rather than pathological confirmation. These limitations highlight the necessity for future multi-center prospective trials to validate [^18^F]mFBG PET/CT against [^123^I]MIBG SPECT/CT, define its role in comprehensive neuroblastoma assessment, and refine imaging protocols across different clinical scenarios.

Among the 35 patients categorized within the high-risk group, 28 exhibited poorly differentiated pathological types. The number of positive cases identified through [^18^F]mFBG PET/CT and [^18^F]FDG PET/CT was 17 and 13, respectively. Within the high-risk cohort, 10 cases demonstrated MYCN amplification, with 5 cases positive for [^18^F]mFBG PET/CT and 4 for [^18^F]FDG PET/CT. In the subgroup characterized by poorly differentiation and MYCN amplification, the difference in positivity rates between [^18^F]FDG PET/CT and [^18^F]mFBG PET/CT was not statistically significant, with P values of 0.125 and 1.00, respectively. Previous researches suggest that patients with poorly differentiated tumors and MYCN amplification were more likely to be MIBG-negative, indicating that they might particularly benefit from [^18^F]FDG PET/CT imaging. However, this study did not observe a higher positivity rate for [^18^F]FDG PET/CT, compared to [^18^F]mFBG PET/CT in patients with poorly differentiated pathological types and MYCN amplification. This finding could be attributed to the limited sample size, alongside the fact that [^18^F]mFBG PET/CT offers higher resolution and sensitivity than MIBG. Moreover, the criteria for high-risk classification encompass more than just differentiation status and MYCN amplification.

Two pediatric patients underwent sequential paired [^18^F]FDG-[^18^F]mFBG PET/CT scans at different therapeutic time points, with each scan pair analyzed independently. The follow-up [^18^F]mFBG PET/CT demonstrated reduced tumor burden (both in lesion number and TBR values) compared to baseline scans, while [^18^F]FDG PET/CT failed to show comparable treatment-related changes. This differential response pattern suggests [^18^F]mFBG’s superior utility for therapeutic monitoring. Furthermore, the technical advantages of [^18^F]mFBG PET/CT - including superior spatial resolution and whole-body coverage - enabled enhanced tissue delineation and more precise anatomical localization of pathological uptake, even in regions adjacent to areas of physiological tracer accumulation (22, 25, 29, 30).

The administration of [^18^F]mFBG in our study demonstrated an excellent safety profile, with all patients tolerating the procedure well - findings that align with previous clinical reports (25, 29, 31). Importantly, the majority of pediatric subjects (particularly valuable in neuroblastoma cases where most patients are <5 years old) successfully completed the imaging procedure without requiring sedation (32–34).

In our protocol, we selected a 1-hour post-injection acquisition time for PET/CT imaging to optimize patient convenience while maintaining diagnostic quality. This decision aligns with existing literature: Pandit-Taskar et al. demonstrated improved lesion detection and tumor-to-background contrast with scans obtained beyond 1-hour post-injection (22), while Atia Samim et al. Reported stable tumor uptake of [^18^F]mFBG between 1–2 hours post-administration, with only minimal background uptake reduction at the later time-point (25).

Our study has several important limitations that should be acknowledged. The most significant constraint is the inability to directly compare our results with the current gold standard [^123^I]MIBG SPECT/CT, primarily due to limited availability of this trace in many regions, especially Southwest China (6, 9, 35, 36). This highlights the urgent need for alternative PET tracers with enhanced diagnostic capabilities. Additionally, the relatively small sample size across different categories (End of Therapy, Follow-up, and Relapse) may introduce chance variations, limiting the reliability of statistical analyses. While [^18^F]FDG PET/CT offers superior spatial resolution and is particularly valuable for MIBG-negative cases, its diagnostic value is compromised by nonspecific bone/bone marrow uptake during post-treatment recovery and intense physiological brain uptake, which can obscure true pathology (14, 15, 37–39). Another potential limitation of [18F]mFBG is its limited availability, as it requires cyclotron production, which may affect broader clinical implementation.

In our study, [^18^F]mFBG PET/CT emerges as promising alternative, sharing the same norepinephrine transporter mechanism as [^123^I]MIBG SPECT/CT while overcoming conventional limitations through PET’s superior spatial resolution and quantitative capabilities (22, 24, 25, 32). However, the interpretation of our findings should consider that most patients (42/56) had undergone prior surgery and chemotherapy, which likely suppressed tumor metabolism and consequently reduced [^18^F]FDG detection rates. To address these limitations, future studies should include more treatment-naïve patients and implement a synchronous trimodality imaging approach ([¹²³I]mIBG/[^18^F]mFBG/[^18^F]FDG) to minimize potential temporal biases and provide more comprehensive evaluations.

## Conclusion

This pilot study highlights the superior diagnostic performance of [^18^F]mFBG PET/CT over [^18^F]FDG PET/CT in neuroblastoma evaluation, suggesting that [^18^F]FDG PET/CT should not be routinely used for this purpose. The significantly higher lesion detection rate with [^18^F]mFBG PET/CT, particularly for skeletal metastases, has critical clinical implications for accurate staging and treatment monitoring. When [^123^I]MIBG scintigraphy is unavailable, [^18^F]mFBG PET/CT emerges as a more reliable alternative than [^18^F]FDG PET/CT. Nevertheless, large-scale prospective trials directly comparing [^18^F]mFBG PET/CT with the current gold-standard [^123^I]MIBG imaging are warranted to validate these findings and potentially redefine standard neuroblastoma imaging protocols.

## Data Availability

The original contributions presented in the study are included in the article/supplementary material. Further inquiries can be directed to the corresponding authors.
